# Ultra-High Refractive Index Sensing Structure Based on a Metal-Insulator-Metal Waveguide-Coupled T-Shape Cavity with Metal Nanorod Defects

**DOI:** 10.3390/nano9101433

**Published:** 2019-10-10

**Authors:** Yuan-Fong Chou Chau, Chung-Ting Chou Chao, Hung Ji Huang, N. T. R. N. Kumara, Chee Ming Lim, Hai-Pang Chiang

**Affiliations:** 1 Centre for Advanced Material and Energy Sciences, Universiti Brunei Darussalam, Tungku Link, Gadong BE1410, Negara Brunei Darussalam; roshan.kumara@ubd.edu.bn (N.T.R.N.K.); cheeming.lim@ubd.edu.bn (C.M.L.); 2 Institute of Optoelectronic Sciences, National Taiwan Ocean University, No. 2 Pei-Ning Rd., Keelung 202, Taiwan; suyang191@gmail.com; 3 Taiwan Instrument Research Institute, National Applied Research Laboratories, Hsinchu 300, Taiwan; hjhuang@narlabs.org.tw; 4 Institute of Physics, Academia Sinica, Taipei 115, Taiwan

**Keywords:** plasmonics, metal–insulator–metal, finite element method, nanorod defects, sensitivity, T-shape cavity, refractive index sensor, temperature sensor

## Abstract

An ultra-high plasmonic refractive index sensing structure composed of a metal–insulator–metal (MIM) waveguide coupled to a T-shape cavity and several metal nanorod defects is proposed and investigated by using finite element method. The designed plasmonic MIM waveguide can constitute a cavity resonance zone and the metal nanorod defects can effectively trap the light in the T-shape cavity. The results reveal that both the size of defects in wider rectangular cavity and the length of narrower rectangular cavity are primary factors increasing the sensitivity performance. The sensitivity can achieve as high as 8280 nm/RIU (RIU denotes the refractive index unit), which is the highest sensitivity reported in plasmonic MIM waveguide-based sensors to our knowledge. In addition, the proposed structure can also serve as a temperature sensor with temperature sensitivity as high as 3.30 nm/°C. The designed structure with simplicity and ease of fabrication can be applied in sensitivity nanometer scale refractive index sensor and may potentially be used in optical on-chip nanosensor.

## 1. Introduction

Surface plasmon polaritons (SPPs) are electromagnetic (EM) waves coherently coupled to electron oscillations which travel at the boundary between a metal and a dielectric [1,2,3], with evanescently decaying EM waves in both borders, which propagate along the metal–dielectric interface [4]. SPP waveguide structures [5,6,7], in particular, metal–insulator–metal (MIM) waveguides [8] with small size, ease of integration, and good balance between light localization and propagation loss [9], have attracted much attention with expectations to realize highly integrated optical circuits because of their behaviour of overcoming the diffraction limit of light [10]. Recently, several plasmonic MIM waveguide sensors have been proposed [11,12] and have been used to the development of sub-wavelength photonic devices such as splitters [13], couplers [14], and filters [15,16,17,18,19,20]. These devices basically consist of waveguides and resonators (or cavities). Because the MIM waveguide sensor is easy to connect with the sensing medium compared to the other sensor structures (e.g., photonic crystal fiber (PCF) sensor [21]) for detecting and sensing applications, many plasmonic MIM waveguide sensor structures have been explored based on surface plasmon resonance (SPR) [22,23] and cavity plasmon resonance (CPR) effects [7,24].

Mid-infrared (MIR) spectrum is in the wavelength range of 2 μm to 20 μm, which represents the molecular fingerprint zone [25], and the potential MIR applications have been widely reported in many works [26,27,28,29,30,31]. In particular, one of the purposes of plasmonic MIM waveguide sensors is necessary for atmospheric transparent window of MIR spectrum from 2 μm to 12 μm [32]. Although there have been reported a number of articles regarding diverse plasmonic MIM waveguides, the interaction nature of incident MIR EM wave and tunable MIM waveguide are investigated less. The necessity of tunable plasmonic MIM waveguide to achieve multifunctionalities in the MIR wavelength range grows into a possible approach for potential applications in chip-scale and integrated plasmonic devices. Plasmonic MIM waveguides necessitate being designed as simple and tuneable in a broadband spectrum if they are to be successfully implemented in sensing applications. The drawback of PCF sensors is that they are not suitable for chip-scale and integrated plasmonic devices. Compared with the PCF sensor [33], the sensitivity of MIM waveguides is much less than that of PCF sensor and still needs to be increased. Therefore, how to improve the sensitivity performance is a key issue in designing plasmonic MIM waveguide sensor.

In this paper, an ultra-high sensitivity of plasmonic structure based on MIM waveguide with a T-shape cavity and several silver nanorod defects compared with the case without the silver nanorod defects has been proposed and investigated. The transmittance spectrum properties of the proposed structure are investigated by means of finite element method (FEM) with perfect matched layers absorbing boundary condition. The sensitivity is calculated to characterize its sensing performance and filter properties. In the proposed MIM waveguide structure, a T-shape cavity is used and several silver nanorod defects are positioned in the T-shape cavity, such that it is approachable to the testing medium and ambient temperature. The T-shape cavity can constitute a resonance source and the silver nanorod defects can clasp the mode size in nanometer scale. The positions of transmission dips show a linear relationship with both RI of the material under RI sensing and surrounding temperature. Sensitivity obtained from the proposed structure reaches 8280 nm/RIU (where RIU is refractive index unit) and 3.30 nm/°C, respectively, which is far greater than the conventional RI sensors and temperature sensors [4,8,15,22,24]. This makes the designed MIM waveguide a promising plasmonic sensor that can provide a route for application in high-density photonic circuits and biosensors.

## 2. Simulation Method and Models

**Figure 1:**
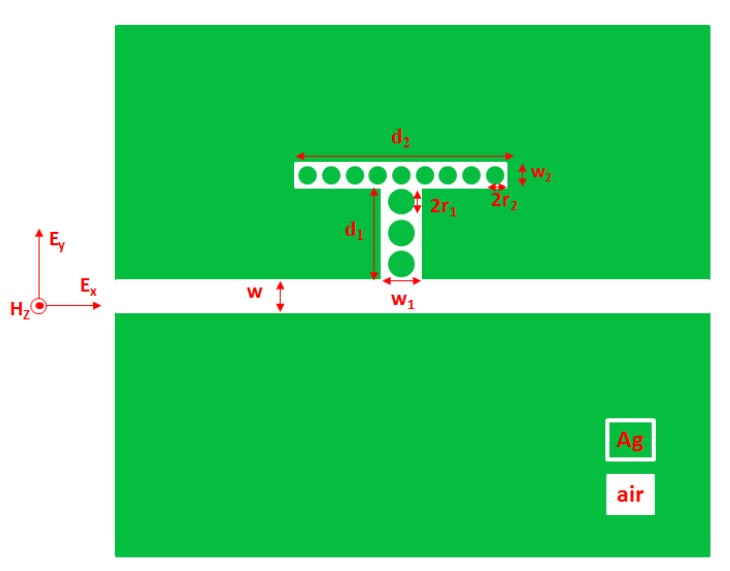
Schematic diagram of the proposed metal–insulator–metal (MIM) sensor structure coupled with several silver nanorod defects in a T-shape cavity.

Figure 1 displays the schematic of a two-dimensional (2-D) plasmonic MIM waveguide structure which consists of a slit (with width *w*), a laterally coupled T-shape cavity and several silver nanorod defects in a T-shape cavity. The T-shape cavity is comprised of a wider rectangular cavity (with width *w*_1_ and length *d*_1_) and a narrower rectangular cavity (with width *w*_2_ and length *d*_2_). Three silver nanorods in wider rectangular cavity (radius *r*_1_) and nine silver nanorods in narrower rectangular cavity (radius *r*_2_) are uniformly distributed in the T-shape cavity. The distance between the center to the center of the adjacent silver nanorod is fixed to be 50 nm. The materials in white and green colors are set to be air and silver as shown in Figure 1. The testing liquid or gas is loaded in the waveguide slit and the T-shape cavity. In the practical situation, the liquid filling can be realized by capillary attraction. The incident EM wave can be coupled into the input port of waveguide by using nano-fiber (e.g., photonic crystal fiber (PCF) [34,35,36]) and the light in the output port of waveguide can be monitored by using Confocal Raman Microscopy [37]. 

The numerical simulation of the designed MIM waveguide was performed by using 2-D FEM (COMSOL multiphysics [38]) with perfect matched layer absorbing boundary conditions at all boundaries of the simulation region. The TM-polarized incident EM wave with inplane electric field components along the *x*-direction is directly coupled to the fundamental SPP mode [39]. Only the TM mode comprising of E*_x_*, E*_y_* and H*_z_* components is considered because of its manifest plasmon phenomenon on the nanometal surface. The transmittance spectrum of the proposed plasmonic MIM waveguide are calculated by parameter scanning the incident wavelength in steps of 1 nm and the transmittance (*T*) is calculated by *T* = *P*_out_/*P*_in_, where the output power *P*_out_ and input power *P*_in_ are defined as integral values of energy flux density, respectively. The stable standing EM wave in the plasmonic MIM waveguide can only make up constructively within the T-shape cavity when the following resonant condition is satisfied [40]:

(1) Δφ=2πn,

where *n* is the mode number which is a positive number (*n* = 1, 2, …).

If the resonance condition in the T-shape cavity is satisfied, the SPPs aroused in the slit would be coupled into the resonant T-shape cavity located next to the slit and develop a standing wave. The resonance wavelength (λ_res_) is given by [7,41]:

(2) $$\lambda_{res}=\frac{2\;Ln_{\mathrm{eff}}}{n-\frac{\varphi_{\mathrm{ref}}}{\pi }},$$

where *L* is the effective length of the cavity (or resonator) and *n*_eff_ represents the real part of effective refractive index (RI) of the SPP, and φ_ref_ is the phase shift of SPP reflection at the cavity metal wall. It can be observed that this has a linear relationship with the cavity length. 

The frequency-dependent complex relative permittivity of silver is taken from [42]. The sensitivity (S) can be calculated as *S* = Δλ/Δ*n* nanometer per refractive index (nm/RIU) [12,24,25,37,40], where Δλ is the shift of resonant peak wavelength of transmittance, λ_res_ is the resonant wavelength and Δ*n* is the RI difference. The width of the slit waveguide (*w*) is kept constant to guarantee that only the fundamental transverse magnetic (TM_0_) mode is excited in the MIM waveguides [43]. The dispersion relation of the fundamental mode (TM_0_) in the proposed plasmonic MIM waveguide can be determined by the equation [44,45,46]:

(3) $$\varepsilon_{d}k_{2}+\varepsilon_{m}k_{1}\tan h\left(\frac{k_{1}}{2}\omega \right)=0\;,$$

where *k_m_* and *k_d_* are defined as: $k_{1}=\sqrt{\beta^{2}-\varepsilon_{d}k_{0}{}^{2}}$ and $k_{2}=\sqrt{\beta^{2}-\varepsilon_{m}k_{0}{}^{2}}$; here ε_m_ and ε_d_ are dielectric constants of the metal and dielectric, respectively. β stands for the constant of propagation for SPPs and $k_{0}=2\pi /\lambda_{0}$ is the wave vector of light with wavelength $\lambda_{0}$ in free-space. The effective RI of the waveguide is denoted as *n*_eff_ = β/*k*_0_. 

## 3. Results and Discussion

**Figure 2:**
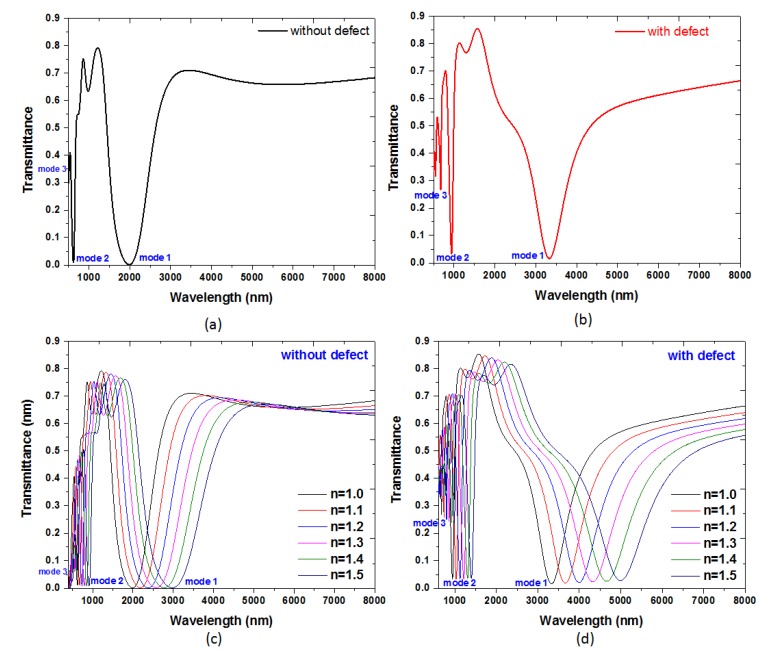
Transmittance spectrum of the proposed plasmonic MIM waveguide (**a**) without defects and (**b**) with silver nanorod defects. Transmittance spectra of the proposed MIM sensor (**c**) without and (**d**) with the silver nanorod defects in the T-shape cavity filled with different refractive index (RI) (*n* = 1.00, 1.10, 1.20, 1.30, 1.40 and 1.50) in the active region of the sensor. The structural parameters of the proposed MIM waveguide, *w*, *w*_1_, *w*_2_, *d*_1_, *d_2_*, *r*_1_, *r*_2_, are set to be 50 nm, 50 nm, 30 nm, 150 nm, 330 nm, 20 nm and 10 nm, respectively.

When the structural parameters of the proposed MIM waveguide, *w*, *w*_1_, *w*_2_, *d*_1_, *d*_2_, *r*_1_, *r*_2_, are set to be 50 nm, 50 nm, 30 nm, 150 nm, 330 nm, 20 nm and 10 nm, respectively, the transmittance spectra of the proposed plasmonic MIM waveguide without defects and without defects in the T-shape cavity are shown in Figure 2a,b, respectively. In Figure 2a,b, it is found that there are three distinct resonance dips occurred at λ_res_ = 1985 nm, 620 nm and 449 nm (i.e., mode 1, mode 2 and mode 3) for the case without defects and at λ_res_ = 3330 nm, 940 nm and 674 nm (i.e., mode 1, mode 2 and mode 3) for the case with detects, respectively. The distinct mode found in the case with defects is arisen from the silver nanorods in the T-shape cavity. When the incident EM wave propagates along the slit of the waveguide, the EM energy can couple to the T-shape cavity, and these transmittance dips are attributed to SPR and CPR modes caused by the coupled SPP, and the transmittance dip approaches to minimum when the resonance condition of the MIM waveguide is satisfied. The depth of transmittance dip depends on the different resonant condition generated in the T-shape cavity. It is worth noting that there is a higher (or a local minimum) transmittance dip between mode 1 and mode 2, i.e., λ_res_ = 979 nm for the case without defects (Figure 2a) and λ_res_ = 1296 nm for the case with defects (Figure 2b). The higher transmittance dip is attributed to the less SPR and CPR effects occurring in the T-shape cavity. In Figure 2a,b, the dip width of mode 1 is wider than those of mode 2 and mode 3, and can be attributed to the interference superposition of the transmitted and reflected EM waves in the T-shape cavity. On the basis of our simulations (the results are not shown here), one can narrow the line width (or increase the Q factor) of mode 1 in various required wavelengths by changing the size of *w*_1_, *d*_1_, *d*_2_ and *r*_1_ in T-shape cavity. 

**Figure 3:**
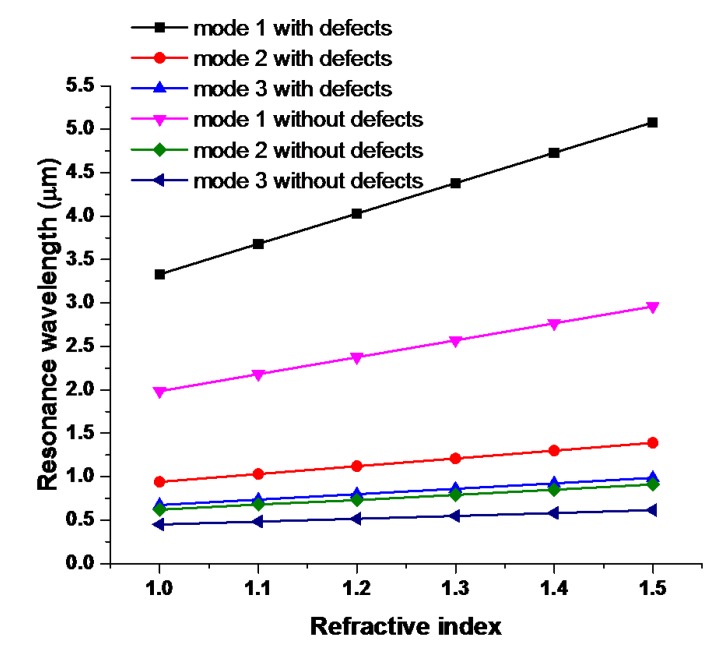
Resonant wavelengths versus the refractive index (RI) with and without silver nanorod defects in T-shape cavity.

Figure 2c,d show transmittance spectra of the proposed MIM sensor without and with the silver nanorod defects in the T-shape cavity filled with different RI (*n* = 1.00, 1.10, 1.20, 1.30, 1.40 and 1.50) in active region of the sensor. The positions of transmittance dips show a linear relationship with RI of the material under sensing. Since the correlation relation between *λ*_res_ and *n*_eff_ obtained from Equation (2), i.e., *λ*_res_ proportional to *n*_eff_, the calculated transmittance spectra of the two cases display a redshift as the increasing RI. According to Figure 3, the RI sensitivities for the case without defects are 1970.0 nm/RIU for mode 1, 560.0 nm/RIU for mode 2 and 330.0 nm/RIU for mode 3, and for the case with defects are 3330.0 nm/RIU for mode 1, 940.0 nm/RIU for mode 2 and 620.0 nm/RIU for mode 3, respectively. Note that the existence of silver nanorod defects in the T-shape cavity leads to increase of device sensitivity of 3330.0 nm/RIU compared to 1970.0 nm/RIU in the case without defects. Namely, the corresponding sensitivity is improved 1.69 times with the existence of silver nanorod defects in the T-shape cavity. 

**Figure 4:**
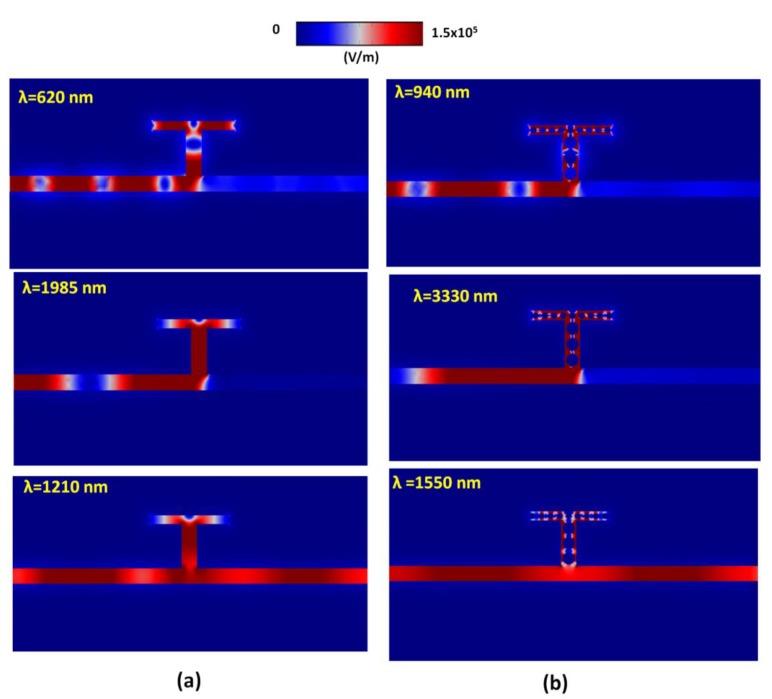
Electric field intensity (|***E***| = (E*_x_*^2^ + E*_y_*^2^)^1/2^) for the cases (**a**) without defects (at *λ*_res_ = 620 nm, 1985 nm and 1210 nm) and (**b**) with defects (at *λ*_res_ = 940 nm, 3330 nm and 1550 nm), respectively.

To further verify the results as shown in Figure 2, we show the electric field intensity (|***E***| = (E*_x_*^2^ + E*_y_*^2^)^1/2^) for the cases without defects (at *λ*_res_ = 620 nm, 1985 nm and 1210 nm, see Figure 4a) and with defects (at *λ*_res_ = 940 nm, 3330 nm and 1550 nm, see Figure 4b), respectively. It is found that the electric field energy of the SPPs in the waveguide is mostly coupled to the T-shape cavity at resonance wavelengths, *λ*_res_. On the contrary, a little electric field energy is coupled to the T-shape cavity, but most propagates directly to the outgoing port of the MIM waveguide at non-resonance wavelengths. The |***E***| profiles show a standing wave pattern in the case without defects and exhibit standing wave like light trapping on the surface of silver nanorods and silver wall of slits in the case with defects due to the gap and edge enhancements [47]. 

**Figure 5:**
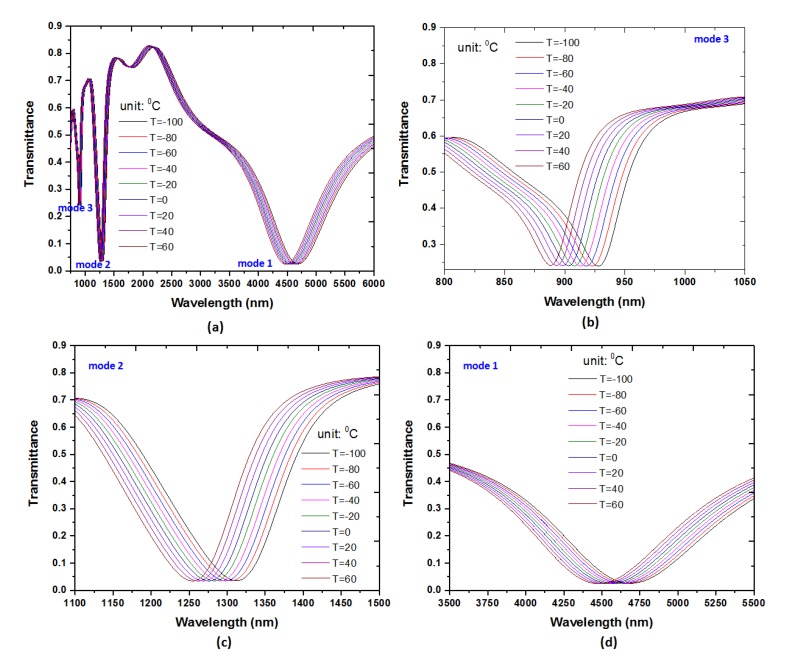
(**a**) Transmittance spectrum of the proposed MIM plasmonic waveguide with different ambient temperature in the wavelength range of 700–6000 nm. Transmittance spectrum of the proposed MIM plasmonic waveguide with different ambient temperature (**b**) in the wavelength range of 888–928 nm for mode 3, (**c**) in the wavelength range of 1254–1312 nm for mode 2, and (**d**) in the wavelength range of 4478–4688 nm for mode 1, respectively. The other parameters are set as the same as used in Figure 2.

The proposed structure is also suitable to be served as a nanoscale temperature sensor [22,37,48] and has the route to compensate temperature for sensor [12,24,49,50,51]. As a temperature sensor, a liquid, ethanol, with high RI temperature coefficient (i.e., d*n*/d*T* = 3.94 × 10^−4^) can be loaded into the slit and T-shape cavity. Thus, the ethanol-sealed cavities in the proposed structure consist of a T-shape resonator and a slit (i.e., a straight waveguide), are sandwiched by two opposite silver walls. The RI of ethanol can be written as [49]:

(4) $$n=1.36048-3.94\times 10^{-4}(T-T_{0}),$$

where *T* is the ambient temperature and *T*_0_ (room temperature) is set to be 20 °C [22]. Equation (4) displays a linear relationship between the *n* and *T.* The sensitivity of temperature sensor is defined as S_T_ = ∆*n/*∆*T*. Figure 5a shows the transmittance spectrum of the proposed MIM plasmonic waveguide with different ambient temperature (T) and the other parameters are kept the same as used in Figure 2. As can be observed from Figure 5a, the λ_res_ shifts to the shorter wavelengths (i.e., blueshift) when the temperature is increased from −100 °C to 60 °C (in steps of 20 °C). There are three transmittance dips in the wavelength range of 888–928 nm for mode 3 (Figure 5b), 1254–1312 nm for mode 2 (Figure 5c) and 4478–4688 nm for mode 1 (Figure 5d), respectively. The positions of transmittance dips demonstrate a linear relationship with RI of the material under ambient temperature. When the temperature varies from −100 °C to 60 °C, the mode 3, mode 2 and the mode 1 shift 40 nm, 58 nm and 210 nm, respectively, resulting in 0.25 nm/°C, 0.3625 nm/°C and 1.3125 nm/°C for the mode 1, mode 2 and mode 3, respectively. To the best of our knowledge, the RI and temperature sensitivity of the proposed plasmonic MIM waveguide is much higher than the previously reported SPPs waveguide sensors and LSPRs sensors [7,12,24,37,48,50,51]. 

**Figure 6:**
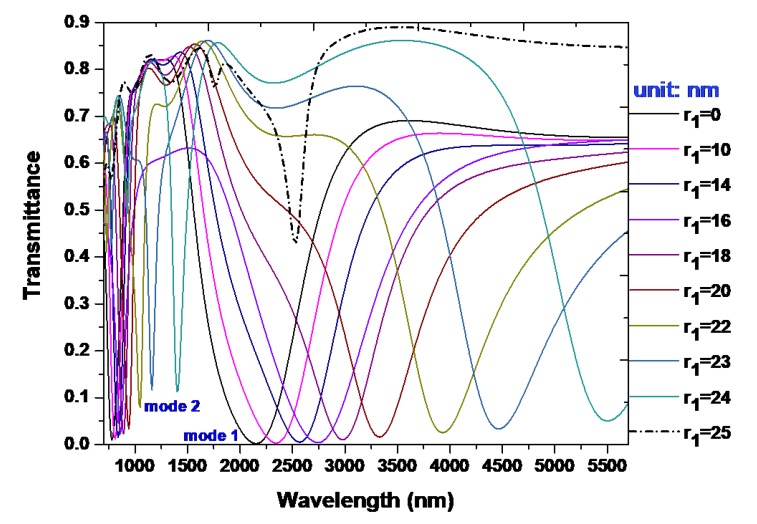
Transmittance spectra for different radius of the silver nanorods with *r*_1_ = (0, 8, 10, 12, 14, 16, 18, 20, 22, 23, 24, 25) nm, respectively. The other parameters are set as the same as used in Figure 2.

The different radius of silver nanorod (*r*_1_) in the wider rectangular cavity will change the resonance condition of free space in the proposed structure, and they have a remarkable influence on the transmittance spectrum. Silver nanorod defects that are positioned at Bragg distance between the silver walls and silver nanorods are composed of a Fabry–Pérot nanocavity [52,53], and they construct a coupled photonic–plasmonic system [54]. In order to study the influence of *r*_1_ in the proposed plasmonic MIM waveguide, the transmittance spectra for different radii of the silver nanorods with *r*_1_ = (0, 8, 10, 12, 14, 16, 18, 20, 22, 23, 24, 25) nm, respectively, were examined (see Figure 6), while *r*_2_ is kept with 10 nm in the narrower rectangular cavity and other parameters are set as the same as used in Figure 2. It is obvious that the *r*_1_ can change the position of transmittance dips evidently, and the wavelengths of the transmittance dips become larger with the increasing of *r*_1_. Table 1 displays three modes and their corresponding calculated sensitivity of the proposed structure under testing medium (*n* = 1.0 and *n* = 1.1) versus the different radius of silver nanorod (*r*_1_). The redshift shift with the increasing *r*_1_ in the range of 0–24 nm as shown in Figure 6 can be explained in terms of the different matching impedance condition at the boundary between the slit and T-shape cavity. Namely, when the *r*_1_ is increased, the changes of *r*_1_ will lead to the impedance match or mismatch. According to the impedance matching condition [55,56,57], the λ_res_ should be increased to guarantee the impedance between the slit and T-shape cavity. 

SPP waves can be sent out and reflected back in the T-shape cavity that can be regarded as a Fabry–Perot cavity, and the resonance condition is reached when the Fabry–Perot condition is satisfied. It is clearly observed from Figure 6 and Table 1 that the *r*_1_ could change the transverse modes due to the different resonant condition being influenced by the defect size that occurred in the T-shape cavity. This implies that the plasmon resonance condition originating from *r*_1_ can be easily tuned by adjusting the gap distance between silver walls and silver nanorods [58]. Therefore, the proposed structure can serve as a good candidate for strong electric field localization and tunable bandpass filters [23].

Here, the plasmon resonance condition is corresponding to the T-shape cavity that is associated with the varied photonic density of states in the T-shape cavity, and hence changed the irradiative damping rate in the proposed structure [59,60]. It is worth noting that a higher transmittance dip (*T* = 42.58%) at λ_res_ = 2528 nm for mode 1 (see the dashed line in Figure 6) is reached when the diameter of the silver nanorod is equal to the width of the wider rectangular cavity, i.e., *w*_1_ = 2*r*_1_ = 50 nm. This is because of the less coupled effect between the slit and T-shape cavity. Through adjusting the *r*_1_, the position of transmittance dip can be tuned linearly in the range of *r*_1_ = (0–24) nm, which is highly advantageous for realizing sensing and wavelength selectivity. In addition, on the basis of our simulations, the varying *r_2_* has much less influence on the enhancing sensitivity compared to the varying *r*_1_. 

**Figure 7:**
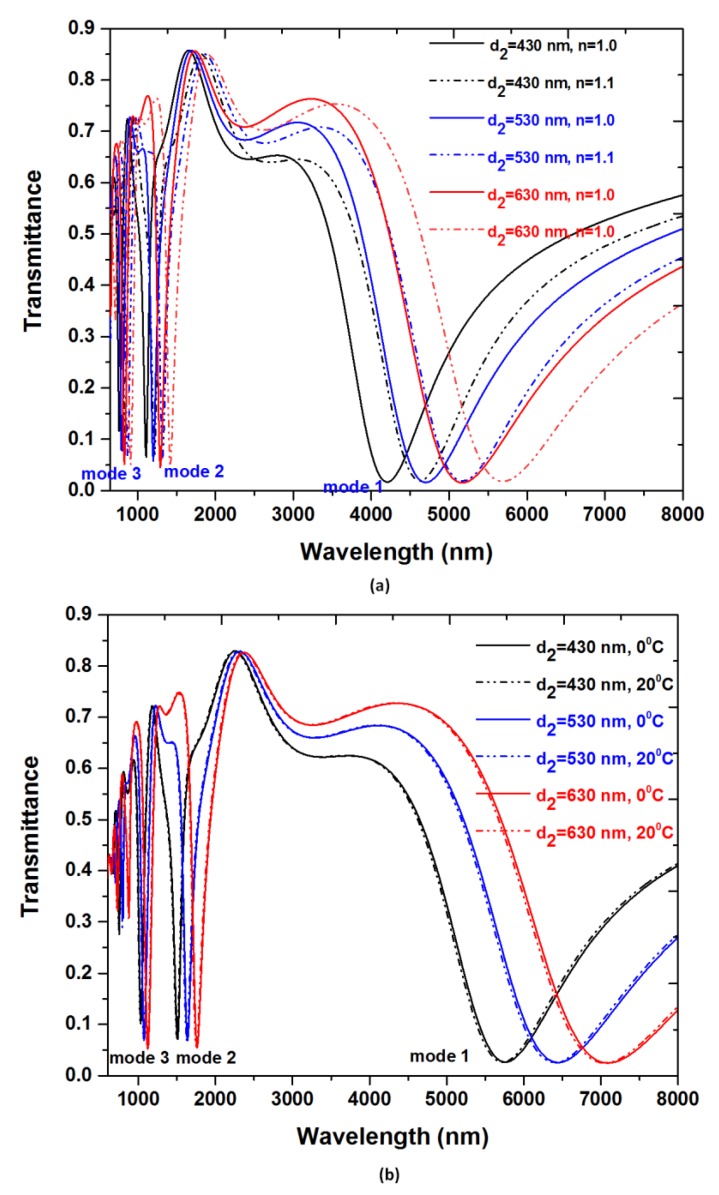
Transmittance spectra as a function of the (**a**) RI (*n* = 1.0 and 1.2) and (**b**) ambient temperature (*T* = 0 °C and 20 °C) for different d_2_ (i.e., 430 nm, 530 nm and 630 nm) of the MIM waveguides. The other parameters are kept the same as used in Figure 2.

Based on Equation (2), the increase of the length (*L*) of the T-shape cavity results in larger λ_res_. Namely, at larger λ_res_, the variation of RI caused by the surrounding medium and ambient temperature has a significant influence on the change of λ_res_. Therefore, RI and temperature sensitivity will be raised. Finally, the influences of the length of narrower rectangular cavity (i.e., *d*_2_) in the proposed structure on sensing sensitivity are analyzed for improving the sensitivity performance. Figure 7 depicts the transmittance spectra as a function of the RI (n=1.0 and 1.2) and ambient temperature (T = 0 °C and 20 °C) for different *d*_2_ (i.e., 430 nm, 530 nm and 630 nm) of the MIM waveguides and other parameters are kept the same as used in Figure 2. While the *d*_2_ is set to be 430 nm, 530 nm and 630 nm, respectively, the RI and temperature sensitivity for mode 1 are 4220.0 nm/RIU and 1.70 nm/°C, 4720.0 nm/RIU and 1.85 nm/°C, 5200nm/RIU and 2.10 nm/°C, correspondingly; the RI and temperature sensitivity for mode 2 are 1080.0 nm/RIU and 0.45 nm/°C, 1190.0 nm/RIU and 0.45 nm/°C, 1270.0 nm/RIU and 0.50 nm/°C, correspondingly; the RI and temperature sensitivity for mode 3 are 720 nm/RIU and 0.30 nm/°C, 750 nm/RIU and 0.30 nm/°C, 790 nm/RIU and 0.30 nm/°C, correspondingly.

According to the results as shown in Figure 6 and Figure 7, we can improve the sensitivity of the SPPs’ waveguide sensor by means of properly tuning both *r*_1_ and *d*_2_ simultaneously. The calculated results are summarized in Table 2 and Table 3 for RI and temperature sensitivity, respectively. Table 2 and Table 3 show three modes and their corresponding RI and temperature sensitivity (S) of the proposed structure with *r*_1_ = 24 nm under testing medium (*n* = 1.0 and *n* = 1.1) and temperature (*T* = 0 °C and *T* = 20 °C) versus different *d*_2_ with 430 nm, 530 nm and 630 nm, respectively. The other parameters are kept the same as used in Figure 2. In Table 2 and Table 3, the λ_res_ of three modes can exist in a wide spectrum range of infrared from 0.97 μm to 1.12 μm. When RI and temperature increase from 1.0 to 1.1 and 0 °C to 20 °C, respectively, the maximum values of sensitivity can reach as high as 8028 nm/RIU and 3.30 nm/0 °C, respectively. To the best of our knowledge, the RI sensitivity of the proposed plasmonic MIM waveguide sensors is much higher compared with previously reported SPPs’ waveguide sensors [22,24,37,40,41,48,59,60,61,62,63,64].

## 4. Conclusions

In this paper, we have proposed an ultra-high plasmonic refractive sensing structure consisting of a MIM waveguide coupled to a T-shape cavity and metal nanorod defects for RI sensing and temperature sensing. The sensing characteristics of the proposed structure are studied by means of 2-D FEM. The designed plasmonic MIM waveguide can constitute a cavity resonance region and the metal nanorod defects can effectively trap the light in the T-shape cavity. The results reveal that the proposed structure possesses three resonant dips in the transmittance spectrum, all of which have a linear relationship under sensing. The maximum values of sensitivity reach as high as 8028 nm/RIU and 3.30 nm/°C, respectively. These results are much higher than those of previously reported SPPs’ waveguide sensors. The designed structure has the merits of compact size, ultra-high sensitive, linear response and large sensing range, which makes it very promising for the application of sensitivity nanometer scale refractive index sensor and enhanced infrared spectroscopy.

## Figures and Tables

**Table 1:** Three modes and their corresponding RI sensitivity of the proposed structure under testing medium (*n* = 1.0 and *n* = 1.1) versus different *r*_1._

*r*_1_ (nm)	Mode 1			Mode 2			Mode 3		
	λ_res_ (nm)		S (nm/RIU)	λ_res_ (nm)		S (nm/RIU)	λ_res_ (nm)		S (nm/RIU)
	*n* = 1.0	*n* = 1.1		*n* = 1.0	*n* = 1.1		*n* = 1.0	*n* = 1.1	
0	2153	2367	2140.0	780	854	740.0	548	593	450
5.0	2197	2416	2190.0	787	862	750.0	551	597	460
8.0	2270	2496	2260.0	798	874	760.0	557	604	470
10.0	2343	2576	2330.0	810	887	770.0	563	611	480
12.0	2440	2683	2430.0	822	900	780.0	573	622	490
14.0	2569	2825	2560.0	838	919	810.0	586	637	510
16.0	2741	3015	2740.0	860	942	820.0	606	660	540
18.0	2978	3275	2970.0	890	976	860.0	634	691	570
20.0	3330	3663	3330.0	940	1031	910.0	674	736	620
22.0	3931	4325	3940.0	1046	1148	1020.0	730	798	680
23.0	4465	4914	4490.0	1160	1273	1130.0	775	847	720
24.0	5502	6056	5540.0	1404	1542	1380.0	923	1010	870
25.0	2528	2778	2500.0	1760	1933	1730.0			

**Table 2:** Three modes and their corresponding RI sensitivity (S) of the proposed structure with *r*_1_ = 24 nm under testing medium (*n* = 1.0 and *n* = 1.1) versus different *d*_2_ with 430 nm, 530 nm and 630 nm, respectively.

*d*_2_ (nm)	Mode 1			Mode 2			Mode 3		
	λ_res_ (nm)		S (nm/RIU)	λ_res_ (nm)		S (nm/RIU)	λ_res_ (nm)		S (nm/RIU)
	*n* = 1.0	*n* = 1.1		*n* = 1.0	*n* = 1.1		*n* = 1.0	*n* = 1.1	
430	6778	7463	6850	1470	1616	1460	998	1095	970
530	7484	8246	7620	1513	1663	1500	1079	1184	1050
630	8146	8974	8280	1563	1717	1540	1154	1267	1130

**Table 3:** Three modes and their corresponding temperature sensitivity (S) of the proposed structure with *r*_1_ = 24 nm under testing temperature (*T* = 0 °C and *T* = 20 °C) versus different *d*_2_ with 430 nm, 530 nm and 630 nm, respectively.

*d*_2_ (nm)	Mode 1			Mode 2			Mode 3		
	λ_res_ (nm)		S (nm/°C)	λ_res_ (nm)		S (nm/°C)	λ_res_ (nm)		S (nm/°C)
	*T* = 0 °C	*T* = 20 °C		*T* = 0 °C	*T* = 20 °C		*T* = 0 °C	*T* = 20 °C	
430	9262	9317	2.75	2006	1995	0.55	1354	1347	0.35
530	10,300	10,239	3.05	2066	2050	0.80	1467	1459	0.40
630	11,214	11,148	3.30	2133	2121	0.60	1570	1562	0.40
